# Metabolism of Melatonin Synthesis-Related Indoles in the Turkey Pineal Organ and Its Modification by Monochromatic Light

**DOI:** 10.3390/ijms21249750

**Published:** 2020-12-21

**Authors:** Kamila Martyniuk, Maria Hanuszewska, Bogdan Lewczuk

**Affiliations:** Department of Histology and Embryology, Faculty of Veterinary Medicine, University of Warmia and Mazury in Olsztyn, Oczapowskiego 13, 10-719 Olsztyn, Poland; kamila.kwiecinska@uwm.edu.pl (K.M.); maria.hanuszewska@uwm.edu.pl (M.H.)

**Keywords:** pineal organ, melatonin, serotonin, indoles, monochromatic light, turkey, birds

## Abstract

The metabolism of pineal indoles is closely related to alterations in the light and dark phases of a daily cycle. Recent research showed important interspecies differences in the pineal biochemistry, and a strong impact of monochromatic light on many physiological processes in birds. Therefore, the aims of study were to characterize the metabolism of melatonin-synthesis indoles in the pineal organ of the domestic turkey, and to determine the changes occurring in this metabolism under the influence of different wavelengths and intensities of light. For this purpose, 3-week-old turkeys were kept under 16 lx white light, or under blue, green, and red light with intensities of 16, 32, and 64 lx during the photophase, and after 7 d were sacrificed at 4 h intervals. The activities of melatonin-synthesizing enzymes and the contents of indoles were measured in the same pineal organ. The results revealed that the activities of tryptophan hydroxylase and arylalkylamine N-acetyltransferase, and the levels of all tryptophan derivatives had significant daily changes in birds kept under each light condition used. The profile of pineal indole metabolism in 4-week-old turkeys was characterized by high-amplitude rhythms in the activity of arylalkylamine N-acetyltransferase and the contents of N-acetylserotonin and melatonin, equal relative amounts of serotonin and 5-hydroxyindoleacetic acid, and higher content of melatonin than N-acetylserotonin. The monochromatic light significantly modified the pineal indole metabolism, and its effects were dependent on the color and intensity of light. Pronounced changes occurred in the level of serotonin synthesis and the daily rhythm course of melatonin synthesis.

## 1. Introduction

Many physiological processes in birds are closely correlated with day and night alterations. The pineal organ and its main hormone, melatonin (MLT), play an important role in regulating these phenomena [1].

Avian pinealocytes are photosensitive due to the presence of two photopigments: pinopsin [2,3] and melanopsin [4]. Pinopsin is specific to the pineal organ, and controls the daily melatonin output [3,5,6]. Melanopsin occurs both in the pineal organ and the retina [7] and is involved in entrainment of the circadian clocks [8]. The axons of melanopsin-containing retinal ganglion cells build the retinal-hypothalamic tract that terminates in the suprachiasmatic nucleus [9,10]. The multisynaptic neuronal pathway connects this nucleus with the pineal organ, both in birds and mammals [11,12].

The color of light has an enormous influence on many physiological functions of birds. Red light reduces feather pecking, aggression, and cannibalism [13], and has a significant effect on egg-laying performance [13,14,15]. Green light increases early age growth of chicken broilers, and blue light increases growth in older birds [16,17,18,19]. Moreover, blue light reduces motor activity [20] and plays a role in alleviating the stress response in broilers [21]. Simultaneous use of green and blue light combines the advantages of these two wavelengths [22]. It has a stimulatory effect on broiler body weight due to significantly improved influence on the feed conversion compared with normal artificial light [23], and enhances immune responses in broilers [21]. Chickens kept under blue light, compared with those reared under green light, attain lower body temperature, and have higher low-density lipoprotein cholesterol and glucose concentrations [24]. The color of light significantly influences the transcription of clock genes in the chicken pineal organ [25,26], however very little is known about the spectral effect of light on MLT synthesis and secretion [27].

Previous studies demonstrated that the pineal organ of the domestic turkey is a very attractive model for chronobiological studies [28,29]. Although both turkeys and chickens belong to the *Galliformes* family, the histological organization and ultrastructure of the turkey pineal organ differ greatly from the chicken pineal organ [30,31,32,33]. The turkey pineal gland possesses a tubule-follicular structure with highly developed rudimentary-receptor pinealocytes, which suggests that direct photoreception plays a primary role in the regulation of melatonin secretion [3,33]. Among all examined domesticated birds, the highest impact of direct pineal photoreception on melatonin secretion was demonstrated in the turkey [28]. The turkey pineal organ very quickly and precisely adapts the secretory activity to the phase-shifts of light–dark cycles and possesses a well-working circadian oscillator.

**Figure A1:**
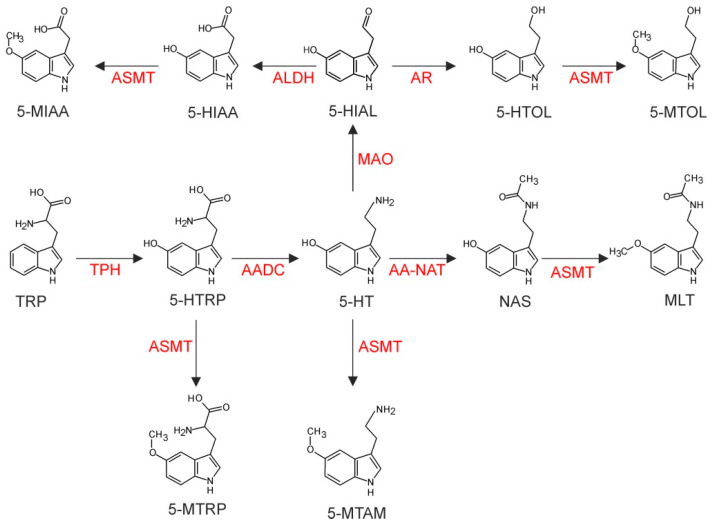
Metabolism of melatonin synthesis-related indoles in the pineal organ. Abbreviations: 5-HIAL, 5-hydroxyindole acetic aldehyde; ALDH, aldehyde dehydrogenase; AR, aldose reductase; MAO, monoamine oxidase; others—see the text above.

Detailed studies on pineal indole metabolism (see Appendix A, Figure A1) based on the measurements of all tryptophan derivatives occurring at considerable levels, have been performed in only a few avian species, including the chicken [34], duck [35], and goose [36,37]. The results showed prominent interspecies differences in the quantitative relationships between indoles and their daily changes in concentration. The obtained data indicate very important species-specific interplays between indoles, which are related to the limiting action of enzymes of the MLT-synthesis pathway. 

The aims of this study were: (i) to characterize the metabolism of MLT-synthesis indoles in the pineal organ of the domestic turkey, and (ii) to determine the changes in this metabolism occurring under the influence of different wavelengths and intensities of light. For this purpose, 3-week-old turkeys were kept under 16 lx white light, or under blue, green, and red light with intensities of 16, 32, and 64 lx during photophases of 7 d, and then euthanized at 4 h intervals. To characterize the indole metabolism, the activities of four enzymes involved in melatonin-synthesis and content of eleven indolic compound were measured in the same pineal organ.

## 2. Results

### 2.1. Activities of Melatonin Synthesis Pathway Enzymes

#### 2.1.1. Tryptophan Hydroxylase

**Figure 1:**
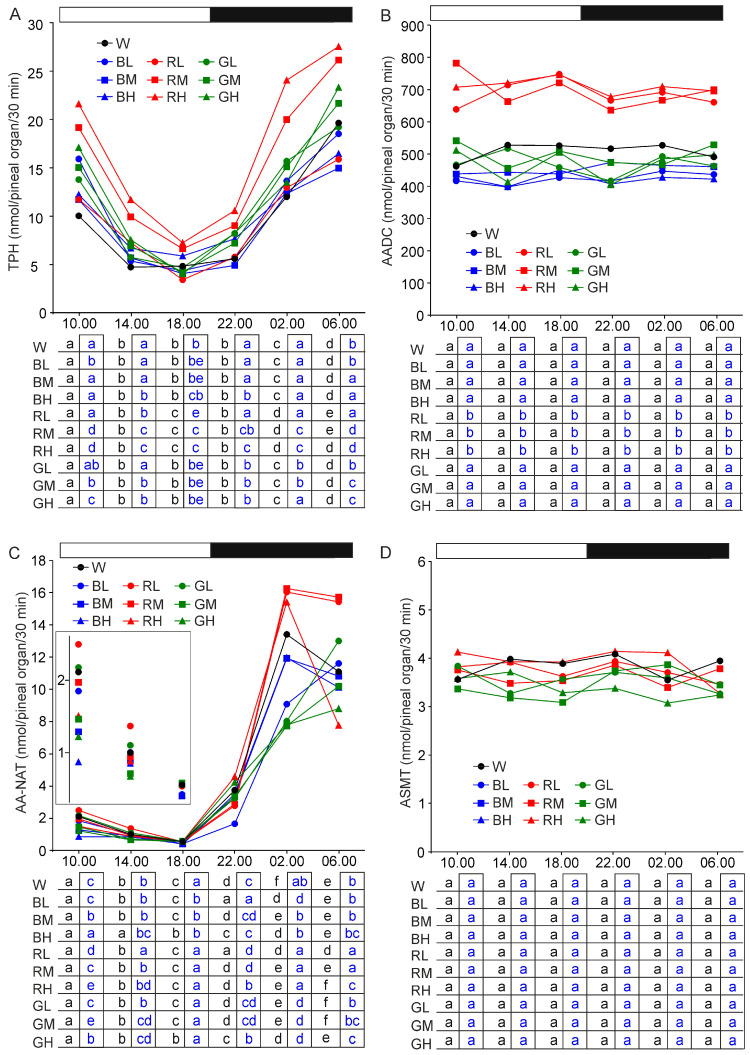
Activity (means) of tryptophan hydroxylase, TPH (**A**), aromatic amino acid decarboxylase, AADC (**B**), arylalkylamine N-acetyltransferase, AA-NAT (**C**) and N-acetylserotonin O-methyltransferase, ASMT (**D**) in the pineal organs of turkeys kept under white light (W) with intensity of 16 lx, blue light with intensities of 16 lx (BL), 32 lx (BM), and 64 1× (BH), red light with intensities of 16 lx (RL), 32 lx (RM), and 64 lx (RH), green light with intensities of 16 lx (GL), 32 lx (GM), and 64 lx (GH) during the photophase. Horizontal bar represents the periods of light and dark phases of a daily cycle. Insert shows data in a limited range of scale. The table charts below report results of the Duncan test. Black letters show differences between time-points within a group (horizontal line) and blue letters - between groups at each time-point (vertical line). The same letters indicate means, which are not significantly different.

The activity of tryptophan hydroxylase (TPH) showed a daily rhythm with significantly higher levels at 02.00, 06.00, and 10.00 than at 14.00, 18.00, and 22.00 in all studied groups of animals (Figure 1A). The difference between peak and nadir was approximately 4 folds. The TPH activity was significantly increased, at all time-points, in animals kept under red light with intensities of 32 and 64 lx, compared with turkeys from other groups. 

#### 2.1.2. Aromatic Amino Acid Decarboxylase 

The activity of aromatic amino acid decarboxylase (AADC) did not change during a daily cycle course in the studied groups of turkeys (Figure 1B). It was significantly higher in birds exposed to red light during the photophase than in control turkeys and animals kept under blue and green lights. There were no differences in AADC activity between turkeys exposed to red illumination with different intensities.

#### 2.1.3. Arylalkylamine N-Acetyltransferase

The activity of arylalkylamine N-acetyltransferase (AA-NAT) showed a prominent daily rhythm, with the lowest level at 18.00 and the highest levels at 02.00 and 06.00, in all studied groups of animals (Figure 1C). The course of daily changes in enzyme activity differed significantly between groups. AA-NAT activity at 10.00, 1 h after the photophase onset, was significantly lower in birds kept under monochromatic light with higher intensity than in those exposed to light with lower intensity. Moreover, at the lowest illumination intensity (16 lx), it was lower in animals kept under blue light than in turkeys exposed to red and green light. At the highest illumination intensity, it was also the lowest in blue-light-exposed animals. Similar differences between the studied groups of animals were also found at 14.00. At 18.00, the enzyme activity was significantly lower in blue-exposed birds than in animals exposed to red, green, and white light. A significant increase in AA-NAT activity was observed at 22.00, after 1 h of darkness. This increase was lower in animals kept under monochromatic light intensity of 16 lx than in those kept under light intensities of 32 and 64 lx. Increase in the enzyme activity was also significantly lower in animals exposed during the photophase to blue light than in turkeys kept under red and green illuminations with the same intensity (except 32 lx). The highest AA-NAT activity was found at 02.00, excluding the groups of birds kept under blue light with intensity of 16 lx and green light with intensities of 16, 32, and 64 lx, in which the peak was noted at 6.00. At 02.00, the highest activity was found in turkeys kept under red light and the lowest activity in animals kept under green light. The differences between peak and nadir of AA-NAT activity rhythm were between 24 and 29 folds in blue, red, and white light exposed animals, and only 13 folds in green exposed birds. 

#### 2.1.4. N-Acetylserotonin O-Methyltransferase 

There were no significant differences in N-acetylserotonin O-methyltransferase (ASMT) activity between the investigated groups of turkeys (Figure 1D). The ASMT activity also did not change significantly in a daily cycle course. 

### 2.2. Content of Melatonin Synthesis-Related Indoles

#### 2.2.1. Tryptophan 

**Figure 2:**
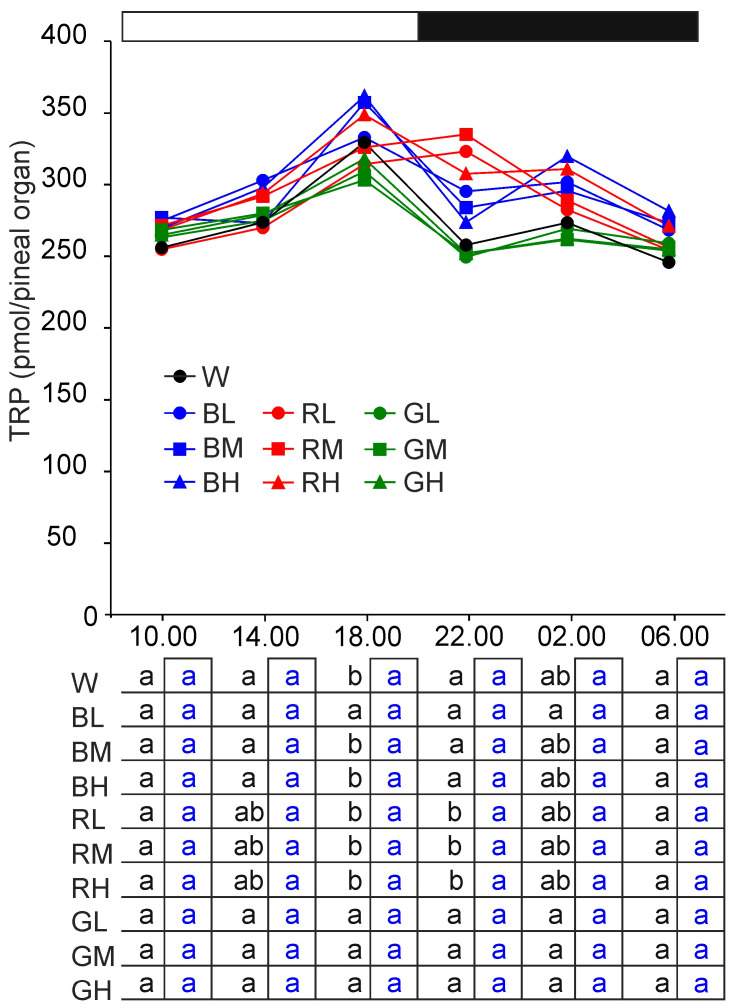
Content (means) of tryptophan (TRP) in the pineal organs of turkeys kept under white light (W) with intensity of 16 1×, blue light with intensities of 16 lx (BL), 32 lx (BM), and 64 lx (BH), red light with intensities of 16 lx (RL), 32 lx (RM), and 64 lx (RH), green light with intensities of 16 lx (GL), 32 lx (GM), and 64 lx (GH) during the photophase. For further explanation, see Figure 1.

The mean content of tryptophan (TRP) varied between 245.8 and 361.8 pmol/pineal organ (Figure 2), but there were large differences in the level of this amine acid between individuals. Two-way analysis of variance (ANOVA) showed no significant effect of illumination (group) and significant effect of sampling time on TRP content. According to the Duncan test, the TRP content was significantly higher at 18.00 than at 10.00, 14.00, 22.00, and 6.00 in control turkeys and animals kept under 32 and 64 lx blue light. It was also significantly higher at 18.00 and 22.00 than at 10.00 and 06.00 in turkeys kept under red light. No significant differences were detected by the Duncan test in turkeys kept under green light and 16 lx blue light. However, it should be noted that the mean values of TRP content in these animals fluctuated in parallel to those in control turkeys. 

#### 2.2.2. 5-Hydroxytryptophan

**Figure 3:**
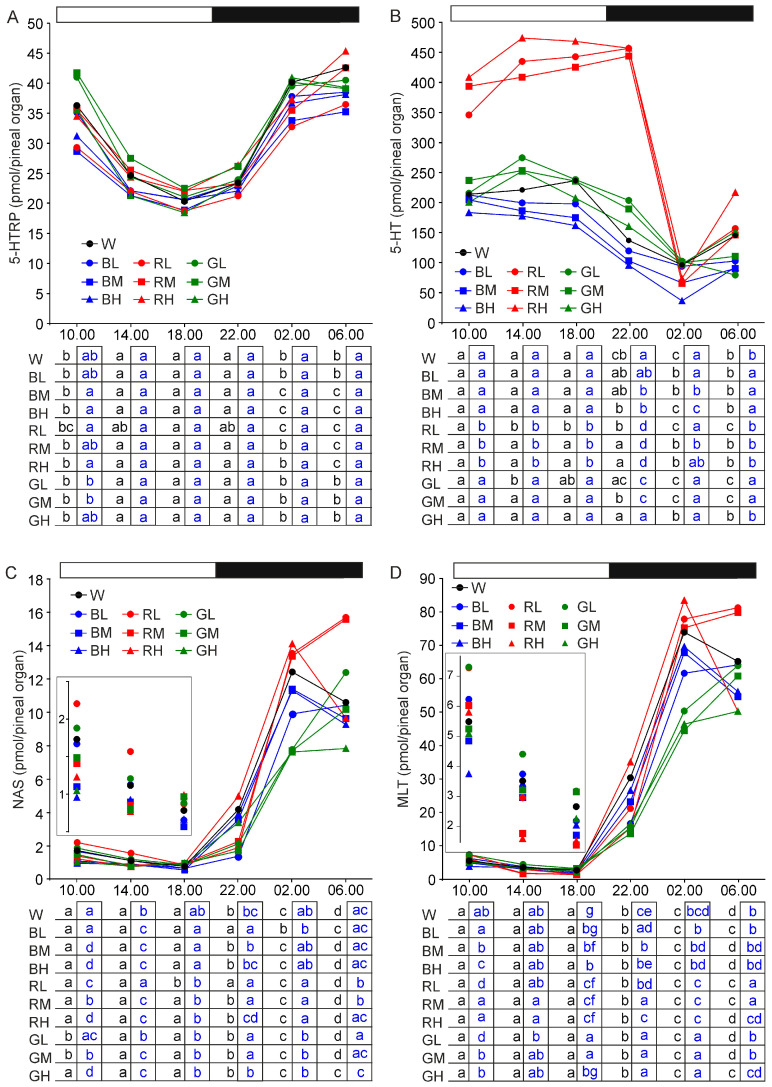
Content (means) of 5-hydroxytryptophan, 5-HTRP (**A**), serotonin, 5-HT (**B**), N-acetylserotonin, NAS (**C**), and melatonin, MLT (**D**) in the pineal organs of turkeys kept under white light (W) with intensity of 16 lx, blue light with intensities of 16 lx (BL), 32 lx (BM), and 64 lx (BH), red light with intensities of 16 lx (RL), 32 lx (RM), and 64 lx (RH), green light with intensities of 16 lx (GL), 32 lx (GM), and 64 lx (GH) during the photophase. For further explanation, see Figure 1.

The content of 5-hydroxytryptophan (5-HTRP) showed a daily rhythm with significantly higher levels at 10.00, 02.00, and 06.00 than at 14.00, 18.00, and 22.00 in all groups of turkeys (Figure 3A). It was also significantly higher at 02.00 and 06.00 than at 10.00 in animals kept under blue light with intensities of 32 and 64 lx, and at 06.00 than at 02.00 and 10.00 in turkeys exposed to red light with intensities of 32 and 64 lx. The difference between the peak and the nadir of the rhythm did not extend 2-fold in all groups of animals. Significant between-groups differences were noted exclusively at 10.00, when the 5-HTRP content was higher in turkeys kept under green light with intensities of 16 and 32 lx than in those kept under blue light.

#### 2.2.3. Serotonin

The serotonin (5-HT) content showed a daily rhythm with significantly higher levels at 10.00, 14.00, and 18.00 than at 02.00 in all studied groups of animals (Figure 3B). Illumination with monochromatic light induced changes in the 5-HT level and the course of daily changes. Red light, independent of its intensity, had the most prominent effect on 5-HT level. The 5-HT content at 10.00, 14.00, 18.00, and 22.00 was approximately 2 folds higher in turkeys kept under red light than in other groups. In the red light exposed animals, the 5-HT content declined by more than 4 folds between 22.00 and 02.00, reaching the level similar to that in other groups. The 5-HT content was significantly higher at 22.00 than at 02.00 (and at 06.00 in most cases) in animals kept under red light, 32 and 64 lx green light, 64 lx blue light, while these differences were not found in animals kept under 16 lx green light, 16 and 32 lx intensity blue light, and white light. The prominent between-groups differences were also observed in the relationship between the content of 5-HT at 02.00 and 06.00. 

#### 2.2.4. N-Acetylserotonin

The content of N-acetylserotonin (NAS) changed significantly in a daily cycle course in all groups (Figure 3C). The nadir value varied from 0.56 to 0.98 pmol/pineal organ and occurred at 18.00, while the peak value varied from 7.89 to 15.69 pmol/pineal and occurred at 02.00 or 06.00, depending on the illumination conditions. The NAS content and its daily changes differed significantly between groups. On the first sampling point during the photophase, at 10.00, the NAS level was higher in animals kept under the 16 lx intensity of red light than blue light. It was also higher after exposure to light with lower intensity than to higher intensity, regardless of color. At 14.00, NAS level was lower than at 10.00 and it was the highest in animals kept under low intensity red light. The between group differences were less prominent at 18.00, except the low NAS level in turkeys kept under blue light. One hour after the onset of darkness, NAS content increased significantly in all groups and this increase was higher in turkey exposed to high intensity light than in those exposed to low intensity monochromatic light during the photophase. At 02.00, NAS content was significantly higher in animals kept under red light than under green light and low (16 lx) intensity blue light. NAS level declined significantly at 06.00 in animals kept under red light with intensity of 64 lx, blue light with intensities of 32 and 64 lx, and white light. In groups with lower intensity of daytime illumination, NAS content increased significantly between 02.00 and 06.00.

#### 2.2.5. Melatonin

The daily changes in MLT content were nearly parallel to those for NAS level, except for higher amplitude of MLT rhythm (Figure 3D). The MLT levels were higher compared with NAS. At the nadir, the mean MLT content varied from 1.38 to 3.15, and at the peak from 50.2 to 83.4 pmol/pineal organ. The between-groups differences in MLT level were also similar to those described for NAS.

#### 2.2.6. 5-Hydroxyindoleacetic Acid and 5-Hydroxytryptophol

**Figure 4:**
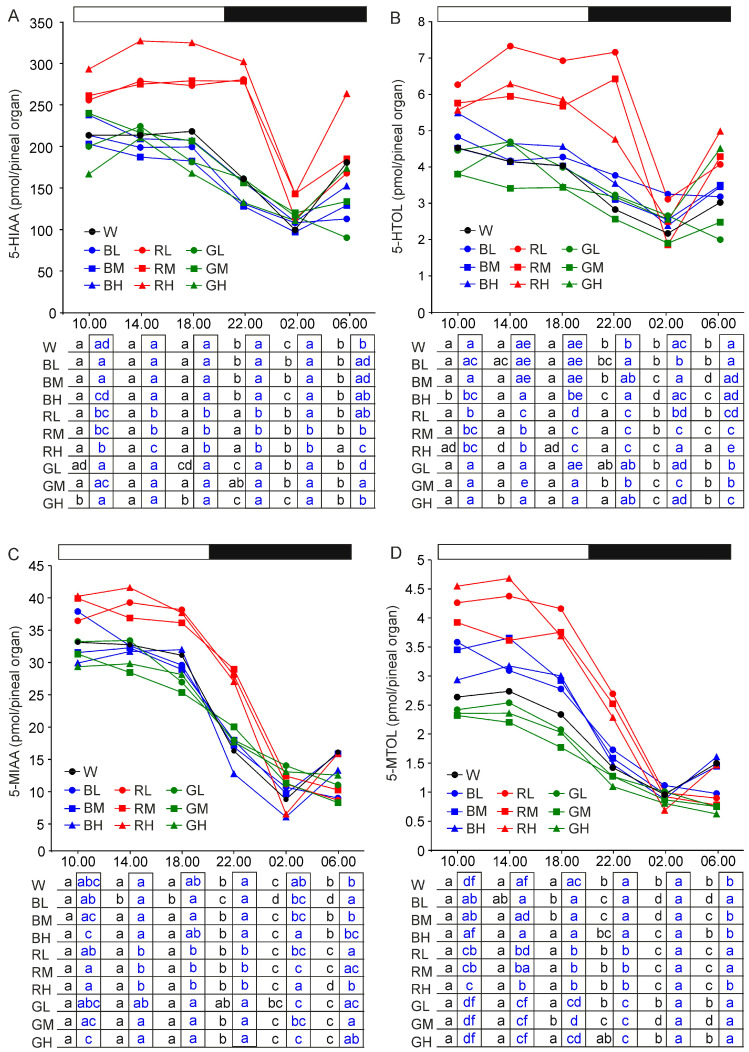
Content (means) of 5-hydroxyindoleacetic acid, 5-HIAA (**A**), 5-hydroxytryptophol, 5-HTOL (**B**), 5-methoxyindoleacetic acid, 5-MIAA (**C**), and 5-methoxytryptophol, 5-MTOL (**D**) in the pineal organs of turkeys kept under white light (W) with intensity of 16 lx, blue light with intensities of 16 lx (BL), 32 lx (BM), and 64 lx (BH), red light with intensities of 16 lx (RL), 32 lx (RM), and 64 lx (RH), green light with intensities of 16 lx (GL), 32 lx (GM), and 64 lx (GH) during the photophase. For further explanation, see Figure 1.

The daily changes and the between groups differences in 5-hydroxyindoleacetic acid (5-HIAA) and 5-hydroxytryptophol (5-HTOL) levels were similar to those described for 5-HT (Figure 4A,B). An exception was the lower proportion between peak and nadir of 5-HIAA and 5-HTOL rhythms compared with 5-HT rhythm in animals kept under red light. The differences in 5-HIAA and 5-HTOL contents between turkeys kept under red light and those from other groups were also smaller than those in 5-HT level.

#### 2.2.7. 5-Methoxyindoleacetic Acid and 5-Methoxytryptophol

The 5-methoxyindoleacetic acid (5-MIAA) content showed a prominent daily rhythm, with the highest level from 10.00 to 18.00, in all groups of turkeys (Figure 4C). At 22.00, the level of 5-MIAA was significantly lower than at 10.00, 14.00, and 18.00. The lowest 5-MIAA level occurred at 02.00 or 06.00, depending on the illumination conditions. 

The 5-MIAA content was significantly higher in turkeys kept under red light than in other animals at 14.00, 18.00, and 22.00. These differences were less prominent at 10.00. At 02.00, the highest 5-MIAA level was noted in turkeys kept under 16 lx and 32 lx green light, and the lowest in 64 lx red light and 64 lx blue light. Similar to 5-HT and 5-HIAA, the 5-MIAA content increased between 02.00 and 06.00 in turkeys kept under 32 lx and 64 lx blue light, 64 lx red light and white light, and slightly decreased in other animals.

The 5-methoxytryptophol (5-MTOL) content was almost 10-folds lower than that of 5-MIAA (Figure 4D). The daily changes in the 5-MTOL level were similar to those described for 5-MIAA, however the between group differences were more prominent. For illustration, the 5-MTOL level at 18.00 and 22.00 was significantly higher in turkeys kept under red light than under blue, white, and green light, and under blue light than under green light. 

#### 2.2.8. 5-Methoxytryptamine and 5-Methoxytryptophan

**Figure 5:**
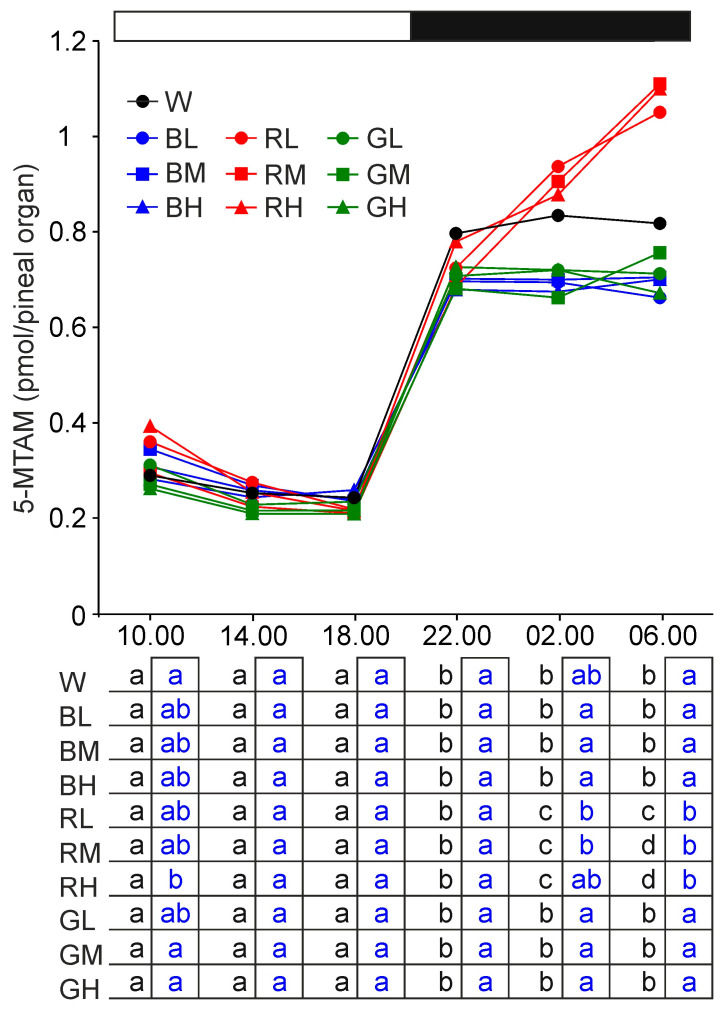
Content (means) of 5-metoxytryptamine (5-MTAM) in the pineal organs of turkeys kept under white light (W) with intensity of 16 lx, blue light with intensities of 16 lx (BL), 32 lx (BM), and 64 lx (BH), red light with intensities of 16 lx (RL), 32 lx (RM), and 64 lx (RH), green light with intensities of 16 lx (GL), 32 lx (GM), and 64 lx (GH) during the photophase. For further explanation, see Figure 1.

The 5-metoxytryptamine (5-MTAM) content showed a daily rhythm with significantly higher levels during the scotophase than during the photophase in turkeys kept under all light conditions (Figure 5). The 5-MTAM content did not change significantly in a course of the scotophase in animals reared under white, blue, and green light. However, in turkeys kept under red light, the 5-MTAM content increased during night and was significantly higher at 02.00 and 06.00 than at 22.00. Consequently, at 02.00 and 06.00, it was higher in red-exposed animals than in other animals exposed to monochromatic light. 5-Methoxytryptophan (5-MTRP) was undetectable in the turkey pineal organ.

## 3. Discussion

### 3.1. Daily Rhythmicity in the Activities of Enzymes Governing Indole Metabolism in the Turkey Pineal Organ

Here, the activity of all four enzymes involved in MLT synthesis and the content of 11 indolic compounds related to this process were measured in the same pineal organ. Such simultaneous and multidimensional measurements were performed for the first time. The obtained results enabled a detailed analysis of changes in the pineal indole metabolism occurring in a daily cycle course. 

Two enzymes, TPH and AA-NAT, showed daily rhythms of their activity in the turkey pineal organ. TPH activity was approximately 4 folds higher at 04.00 and 06.00 than at 14.00, 18.00, and 22.00, and this night-time increase obviously ensured delivery of 5-HT for intensive synthesis of NAS during darkness. The activity of TPH remained significantly elevated at 10.00 enabling renewal of 5-HT pool in pinealocytes. 

In contrast to TPH, AA-NAT activity showed huge daily variations in the turkey pineal gland and these variations were responsible for daily rhythms in the content of all derivatives of TRP, except for 5-HTRP and 5-MTAM. In turkeys kept under white light, the peak value, occurring at 02.00 exceeded the nadir value, occurring at 18.00, approximately 25 folds. The daily changes in AA-NAT activity were reflected in the daily rhythms of NAS and MLT contents. The increase in AA-NAT activity at night resulted also in intensive utilization of 5-HT, and significant decrease in the level of this amine and the levels of the indoles derived from 5-HT oxidative deamination (5-HIAA, 5-HTOL, 5-MIAA, and 5-MTOL). The high amplitude daily fluctuations in the activity of AA-NAT and in the contents of NAS and MLT found in this study concur with our previous data showing the prominent rhythms in the secretion of MLT from the turkey pineal organ in superfusion culture [28] and in the MLT concentration in blood plasma [29].

The activity measurements of an enzyme in tissue homogenate may not reflect the real activity of that enzyme in living cells, therefore their results should be considered cautiously. However, the joint analysis of data concerning enzyme activities and contents of indoles leads to some important remarks. In the pineal organs of 4-week-old turkeys, the intracellular activity of AADC seems to be very high in relation to the substrate level. This idea is derived from the following data: (i) the AADC activity measured in the homogenate is almost 20 folds higher than the TPH activity assayed in vitro, (ii) the ratio of 5-HTRP content to 5-HT content is extremely low, and (iii) the amplitude of daily rhythm of 5-HTRP content is much lower than that of TPH activity rhythm. It should be also noted that decarboxylation into 5-HT is the only identified metabolic pathway of 5-HTRP in the turkey pineal organ, because there were no detectable amounts of 5-MTRP, a compound formed by the methylation of 5-HTRP. 

Special attention should be paid to ASMT activity in the turkey pineal organ, since the NAS content was very low compared with MLT. Additionally, the amplitude of daily changes in NAS level was lower than that of MLT. In the studies performed on the pineal organs of other species, except the chicken, the NAS content was usually much higher than that of MLT, and the amplitude of NAS rhythm was prominently higher than that of MLT [36,37,38]. These relationships between NAS and MLT are commonly considered as evidence for the limiting role of ASMT in the pathway of MLT synthesis [38,39]. Our data suggest that ASMT activity is high enough to ensure the rapid conversion of NAS to MLT even at the nighttime peak, and this enzyme has a very weak or non-limiting effect on MLT synthesis in the pineal organs of 4-week-old turkeys.

In contrast to MLT synthesis, ASMT had a limiting effect on 5-MIAA and 5-MTOL formation at 02.00 and 06.00 in the investigated pineal gland, as shown by the more prominent nocturnal decrease in the 5-MIAA and 5-MTOL contents than in the 5-HIAA and 5-HTOL levels. This could be explained as the effect of very high nocturnal increase in NAS level and much higher affinity of ASMT to NAS than to 5-HIAA and 5-HTOL.

### 3.2. Metabolic Profile of Pineal Indoles

This study characterized for the first time the metabolic profile of MLT synthesis-related indoles in the turkey pineal organ. The previous investigations performed on the pineal organs of geese [36,37], ducks [35], and chicken [34] showed large interspecies differences in quantitative relationships between these indoles among birds. The most prominent of these involved 5-HT and the products of its oxidative deamination. The pineal organs of geese and ducks were characterized by high relative amounts of 5-HT, prominent daily changes in the level of this amine with a decline late in the night and low relative amounts of 5-HIAA. The goose pineal organ had the highest share of 5-HT, which represented 35–60% of all studied indoles (the same indoles were assayed in all cited works). 5-HIAA comprised only 3.0–5.3% of the pineal indoles. In the duck pineal organ, 5-HT represented 15–35% of indoles, depending on the time of day, while 5-HIAA represented 8–15% of indoles. Contrarily, 5-HT represented less than 5% of all investigated indoles in the chicken pineal organ, while 5-HIAA represented 18–38% of the investigated indoles. The ratio of 5-HIAA to 5-HT levels was about 10 in chicken, but below 0.7 in ducks, and 0.1 in geese. The present study showed that 5-HT represented from 15.8% of the investigated indoles at 02.00 to 28.0% at 18.00 in turkeys kept under white light. The ratio of 5-HIAA/5-HT was close to 1 in the turkey pineal organ. Until now, we considered the high relative content of 5-HT as an attribute of the pineal organs of *Anseriformes* birds. This study shows similarities in the relative amount of 5-HT and 5-HIAA/5-HT ratio between the turkey and the duck, and prominent differences between the turkey and the chicken, two species belonging to the same order, *Galliformes*. 

The prominent differences in the pineal indole profiles between the investigated avian species concerns also 5-MTAM. This amine was undetectable or hardly detectable in the chicken pineal organ [34], while it occurred at considerable levels in the pineal organs of the duck [35], the goose [36], and as shown here, the turkey. 

The content of all investigated derivatives of TPR showed significant daily changes in the turkey pineal organ. The nocturnal increase in 5-HTRP level in turkeys was lower than in chicken [34] and close to that in ducks [35] and geese [36]. The important differences among species also concern the time of nocturnal increase in 5-HTRP level, which occurred later in the turkey than in other investigated birds. The 5-HT content showed a large decline in the second part of the scotophase in turkeys, geese, and ducks, while it suddenly increased at the end of night in chicken. The differences between peak and nadir of the daily rhythm of NAS and MLT were much higher in turkeys than in ducks [35] and goose [36], but lower than in chicken [34]. The rhythms of 5-HIAA and 5-HTOL followed that of 5-HT in all investigated species, however large interspecies differences concerned 5-MIAA and 5-MTOL. The daily rhythms of 5-MIAA and 5-MTOL in the duck pineal organ were almost parallel to the daily changes in their immediate precursors [35]. In contrast, the daily rhythms of 5-MIAA and 5-MTOL in the chicken pineal gland had no prior background on the changes in 5-HIAA and 5-HTOL levels [34]. It had been proposed that the extremely large increase in the NAS level at night, together with the much higher affinity of ASTM to this indoleamine than to 5-HIAA and 5-HTOL, decrease the synthesis of 5-MIAA and 5-MTOL. The proposed mechanism explains the occurrence of daily rhythm in 5-MIAA and 5-MTOL levels without analogous changes in the contents of 5-HIAA and 5-HTOL. In turkeys, the rhythms of 5-MIAA and 5-MTOL had higher amplitudes than those of 5-HIAA and 5-HTOL. Like in the chicken pineal organ, this phenomenon seems to have its source in competition among 5-hydroxyindoles for O-methylation by ASMT. Similar to ducks [35] and geese [36], the 5-MTAM level showed a daily rhythm with nocturnal increase in the pineal organ of turkeys. The mechanism responsible for its generation is unknown.

### 3.3. Effect of Monochromatic Light on Pineal Indole Metabolism 

The obtained results showed prominent daily fluctuations in the pineal indole metabolism in birds kept during the photophase under blue, red, and green monochromatic light. First, it should be emphasized that high-amplitude rhythms in the AA-NAT activity and the NAS and MLT contents were found both in turkeys exposed to blue light and in animals exposed to red light, even at the lowest intensity. Melanopsin and pinopsin, two photopigments involved in the reception of light for the entrainment of biological rhythms, show very high sensitivity to blue light and very low sensitivity to red light [2,6,8]. Therefore, blue light is considered a crucial factor in the regulation of daily rhythmicity, and contrary, red light is believed to have very weak or no influence on the daily rhythmicity and it is commonly used as an equivalent of darkness in chronobiological experiments. Yadav et al. [40] demonstrated that the blue and red light periods of a daily cycle were interpreted by birds as day and night, respectively. We supposed that red light with intensity of 16 lx will be too insufficient for maintaining the daily rhythmicity in turkeys, and prominent decreases in amplitude of day-night variations in AA-NAT activity as well as NAS and MLT contents were expected. Contrary to our hypothesis, the obtained data showed that the red illumination with intensity of 16 lx during the photophase is enough for preserving high amplitude daily rhythm in MLT synthesis in turkeys. It should be stressed that in our experiment, birds were kept under complete darkness during the scotophase, which may increase effectivity of dim light in the control of pineal rhythmicity. Recent studies show the significance of darkness at night in many aspects of animal physiology [41].

The monochromatic light significantly modified the pineal indole metabolism in turkeys and its effects were dependent on the color and intensity of the light. The prominent changes occurred in the course of daily rhythm of AA-NAT activity and, consequently, in MLT synthesis. The differences in light conditions affected: (i) the decline in AA-NAT activity after the photophase onset and the level of enzyme activity at the nadir, (ii) the increase in AA-NAT activity after the scotophase onset, and (iii) the time of the peak occurrence and the activity level at the peak. The decline in AA-NAT activity after the photophase onset was significantly higher in turkeys kept under blue light than under green and red light. Moreover, the inhibitory effect of light increased with the illumination intensity, independent of the light color. At the nadir, the AA-NAT activity was the lowest in blue-exposed birds. The increase in enzyme activity after the photophase onset was lower in the animals exposed to blue light than in the turkeys kept under red and green illuminations with the same intensity. The size of this rise increased with the monochromatic light intensity. The peak of AA-NAT activity occurred at 02.00 in turkeys kept under red light and blue light with intensity of 32 and 64 lx, but at 06.00 in birds kept under green light and 16 lx blue light. At the peak, the highest activity was found in turkeys kept under red light. 

The red light significantly affected the performance of enzymes involved in 5-HT synthesis, increasing the activities of TPH and AADC. This effect was prominently reflected in the 5-HT content, which at 10.00, 14.00, 18.00, and 22.00 was approximately 2 folds higher in turkeys kept under red light than in other groups, and in the 5-HIAA, 5-HTOL, 5-MIAA, and 5-MTOL levels. The differences in 5-HIAA and 5-HTOL levels between red-exposed animals and other birds during the photophase were lower than those found in 5-HT level. It seems that the activity of monoamine oxidase is too low to produce more 5-HIAA and 5-HTOL. Interestingly, red light did not change the 5-HTRP level and this phenomenon could be explained by rapid transformation of 5-TRP into 5-HT. The red light affected the course of daily rhythm of 5-MTAM, increasing the level of this amine in the second part of the night. 

The mechanisms by which the spectral light influences the pineal activity remain unknown. Three putative routes of color-dependent light action on the avian pineal organ should be considered: (i) via pinopsin- or melanopsin-positive structures in the pineal organ, (iii) via outer segments of retinal photoreceptor cells, and (ii) via melanopsin-containing processes of retinal ganglion cells. The first option is supported by the results of the in vitro studies showing that the entraining effect of light on MLT secretion is wavelength-related [42]. It should also be stressed that the pineal organ of 4-week-old turkeys comprises extremely well-developed rudimentary photoreceptor pinealocytes with the apical protrusions containing pinopsin [33]. Moreover, recent studies suggested that pinopsin is responsible for highly sensitive light reception in the retinas of lower vertebrates, which points to its ability to detect low-intensity light [43]. Regarding the second option, the retinal photoreceptor cells are good candidates because they are color selective and highly light sensitive. Moreover, they form synaptic contacts with melanopsin-containing ganglion cells. It has been demonstrated that monochromatic light affects the rhythmic expression of clock genes in retina photoreceptor cells in vitro [44]. For the third option, melanopsin-containing processes of retinal ganglion cells, the light spectral sensitivity of these cells needs verification in an in vivo model, to determine their ability to respond to red light. 

It is well-documented that the use of monochromatic light during the photophase changes the circadian and daily rhythms in the expression of clock genes in the avian pineal organ [25,45,46,47,48]. In chicks reared under monochromatic light with intensity of 15 lx and then kept in continuous darkness, the transcription patterns of clock genes were significantly affected by the color of light [25]. The amplitudes and mesors of circadian rhythms were elevated for the positive clock genes (*Clock*, *Bmal1*) and were reduced for the negative clock genes (*Per2*, *Cry*) in green light, and the opposite pattern was observed in red light. Compared with white light, blue light advanced the acrophases of the negative factors and delayed those of the positive elements. In contrast, red light advanced all the clock genes except *cClock* and *cPer2*, while green light delayed all the clock genes except *cBmal2*. The increased mRNA levels of the positive clock genes and the reduced mRNA levels of the negative clock gene were observed in chicks reared under green light but not in red light [25]. The published data on the effect of wavelength on the transcription of clock genes in chicken were subject to advanced statistical analysis by Yang et al. [45]. According to the results, clock gene transcription shows a circadian rhythm in long-wavelength light, while it is arrhythmic in short-wavelength light. *Clock* expression declines during the day and elevates during the night in birds treated with red light, shows an opposite pattern under green light, and is arrhythmic in blue light. Under the influence of green light, *Bmal1* expression elevates during the day and declines at night. However, in birds treated with red and blue light, *Bma1l* expression declines during the day and elevates at night. 

Monochromatic light also affects AA-NAT gene expression in chicks kept under 12/12 L/D cycle [48]. It has been shown that green light enhances AA-NAT mRNA level, while red light decreases the level of this mRNA. The mesors and amplitudes of the daily rhythm of AA-NAT mRNA were higher in green light and lower in red light. Moreover, monochromatic light illumination affected the clock proteins in the chicken pineal gland. The protein levels of CLOCK and BMAL1 were elevated in green light and decreased in red light. Monochromatic light also affects the expression of opsins [48]. 

In contrast with the gene level, knowledge on the effects of monochromatic light on MLT synthetizing enzymes and indole levels is only fragmentary. It has been demonstrated that plasma MLT level during the subjective day under continuous darkness was significantly decreased in chicks reared in red light during the photophase, increased in green light, and was not changed in blue light [25]. Jin et al. [46] showed that green light during the photophase significantly increased the nocturnal plasma melatonin level in 7-day-old chicks. It has also been demonstrated that during the embryonic period, the rhythmic output of melatonin in the chick pineal gland is sensitive to the different monochromatic light wavelengths [47].

The photometric illuminance, expressed in lx, is commonly used as a measure of light intensity in the research of monochromatic light effects in animals, including the present one. The advantage of this measure is an easy comparison of results between studies and with zootechnical standards, because of the popularity. However, the big disadvantage of the photometric illuminance is its reference to the human eye sensitivity at daytime [49]. The spectral sensitivity of animals may differ from that in humans [49,50,51]. Such differences were reported in chickens and turkeys, therefore alternative units called chicken-lx and turkey-lx were proposed [50,51]. Due to differences between human and chicken spectral sensitivity, hens perceive an incandescent light source as 10–20% brighter than a fluorescent light source, when these are measuring the same lux-values [52]. In order to understand the biological effects of light, it is crucial to consider the perceived illuminance of light source by the particular species or even by the particular system in the body. It should be also noted that the measurement of photometric illuminance provided by white and color LED required a dedicated meter to avoid erroneous data. As an alternative for the photometric illuminance, Grubisic et al. [49] recommended measurement of the spectral irradiance (in W/m^2^ × nm), which is much more precise.

## 4. Materials and Methods

### 4.1. Birds and Experimental Design

The study was performed on females of the domestic turkey, Hybrid Converter strain (Hendrix Genetics, Boxmeer, The Netherlands). Due to technical reasons, the experiment was divided into 3 parts: red color, blue color, and green color. Each part started with transporting 1-day-old chicks from a commercial hatchery (Grelavi, SA, Olsztyn, Poland) to the chronobiological animal laboratory at the Faculty of Veterinary Medicine, University of Warmia and Mazury in Olsztyn, Poland, where the turkeys were reared for 4 weeks in controlled temperature and humidity conditions according to zootechnical standards. The birds had free access to standard food and tap water.

The turkeys were kept under a cycle of 12/12 h light/dark (12L:12D). During the first 3 weeks, the light was provided by tungsten lamps (3200 K), which were automatically switched on at 09.00 and switched off at 21.00 of the daily time. The light intensity, measured at a distance of 20 cm from the floor, was 16 lx. After this period, birds were randomly divided into four separate rooms with different light conditions as described in Table 1. The light on and off times remained unchanged. The turkeys kept under white light were considered as the control.

The monochromatic light was provided by water and dust proof modules comprising three red, green, or blue light emitting diodes (GOQLED, Namyangju, Korea) with specifications presented in Table 1. The modules were mounted at equal distances from each other to white polystyrene plate with dimensions 100 × 200 cm (50 cm distance from the room wall on each side), which was suspended 2 m above the floor level. The light intensity was set up by the number of red, blue, and green modules mounted to the plate, and fine adjusted by regulating the supply voltage using the laboratory DC power supply unit (UTP 1305, Dongguan, Uni-Trend Technology, China). The light intensity was measured at a distance of 20 cm from the floor by a light meter dedicated to color LED light measurements (Multi-LED Light Meter TM-209M, Tenmars Electronics, Taipei, China).

The birds were kept in the new light conditions for 7 d. At the age of 4 weeks, six birds from each color/intensity group were sacrificed at 10.00, 14.00, 18.00, 22.00, 2.00, and 6.00. In each part of the experiment, four turkeys kept under white light were euthanized at the same time-points and the samples from these animals established a joint group (*n* = 12). The pineal organs were quickly removed and frozen at −75 °C. During the photophase, the pineal organs were collected under red light with intensity below 5 lx in the red part of the experiment, under green light with intensity below 5 lx in the green part of the experiment, and under blue light with intensity below 5 lx in the blue part of the experiment. An exception was the pineal organs of turkeys kept under white light, which were collected during the photophase under white light with intensity below 5 lx. During the scotophase, the euthanasia of animal was performed under complete darkness and the pineal organs were collected under red light with intensity not exceeding 3 lx in all parts of the experiment. All experimental procedures on birds were performed in accordance with Polish and EU law (AWG FVM UWM in Olsztyn opinion for project NCN ID 448523, 2 December 2019).

The results obtained in the animals kept under white light in the red, blue, and green parts of the experiment were compared with each other to check if there were any significant variations between these parts. This comparison showed no significant differences in activities of all studied enzymes and content of all studied indoles between the parts of the experiment.

### 4.2. Assay of Enzyme Activity and Indole Content

#### 4.2.1. Chemicals

Methanol of gradient-grade high pressure liquid chromatography (HPLC) purity, ammonium iron (II) sulfate hexahydrate, and perchloric acid were provided by Merck Millipore (Billerica, MA, USA). Sodium acetate, sodium dihydrogen phosphate, sodium monohydrated phosphate, disodium EDTA, and acetic acid were purchased from J. T. Baker Chemicals (Center Valley, PA, USA). Tetrahydrobiopterin, dithiothreitol, 3-hydroxybenzylhydrazine dihydrochloride (NSD-1015), catalase from bovine liver, TRIS base, pyridoxal 5’-phosphate hydrate, pargyline, 5-tryptamine, acetyl-CoA sodium salt, S-adenosylmethionine, TRP, 5-HTRP, 5-HT, NAS, MLT, 5-HIAA, 5-MIAA, 5-MTOL, and 5 MTAM were obtained from Sigma-Aldrich (St. Louis, MO, USA). 5-HTOL and 5-MTRP were purchased from Santa Cruz Biotechnology (Dallas, TX, USA). Ultrapure water (18.2 MΩ, TOC ≤ 3 ppb), which was freshly prepared using a Milli-Q^®^ IQ 7003/05 purification system (Merck Millipore, Billerica, MA, USA), was used in all procedures.

#### 4.2.2. Sample Preparation for the Assays of Enzyme Activity and Indole Content

The frozen pineal glands were weighed and sonicated (5 × 2 s, 1 W) in 200 μL of cold water using a Vibra-Cell VC 70 ultrasonic processor equipped with a 2-mm probe (Sonics and Materials Inc., Newtown, CT, USA). Next, 80 μL of homogenate was added to an Eppendorf tube containing 20 μL of 1 mM perchloric acid, the mixture was vortexed, incubated for 15 min in an ice-bath and centrifuged at 30,000× *g* (4 °C) for 15 min (Allegra 64R, Coulter Beckman, Indianapolis, IN, USA). The supernatant was carefully transferred into autosampler vials and used in the assay of melatonin-synthesis related indoles. From the remaining part of the homogenate, 20 μL aliquots were added to Eppendorf tubes containing substrates used in the assays of TPH, AADC, AA-NAT, and ASMT. The time between the tissue sonication and beginning of incubation in the assays of enzymes did not extend beyond 30 s.

#### 4.2.3. Content of Melatonin-Synthesis Related Indoles

The content of indoles in the pineal organ homogenates was measured by HPLC with fluorescence detection [35] using a Vanquish Duo U/HPLC system equipped with two gradient pumps, cooled dual split autosampler, column compartment and two fluorescence detectors (Thermo Fisher Scientific, Waltham, MA, USA). The separation was performed on Hypersil Gold aQ columns with 3 μm particle size and dimensions of 150 × 4.6 mm (Thermo Fisher Scientific, Waltham, MA, USA) at 30 °C. The mobile phase was prepared through on-line mixing of gradient-grade HPLC-purity methanol and a water solution of 5 mM sodium acetate and 0.01 mM disodium EDTA with pH 4.5 (by adding acetic acid). The initial concentration of methanol was 3% (*v*/*v*). Between the 7th and 20th min of the separation, the methanol content was linearly increased to 30% (*v*/*v*) and then maintained at a constant level. The flow rate of the mobile phase was 1 mL/min. The injection volume was 20 μL. The detection was performed at an excitation wavelength of 280 nm and an emission wavelength of 345 nm, at a constant temperature of 38 °C. The sensitivity of the detector was changed at the 15.25th min of the separation from level 6 to 8. The samples were stored in an autosampler at 10 °C for no longer than 8 h. The chromatograms were analyzed using Chromeleon 7.2.10 software (Thermo Fisher Scientific, Waltham, MA, USA). The limits of quantification (S/N ratio of 10:1 and RSD ≤ 15%) for all indoles was below 2 pg per injection. The intra-day precision (RSD of peak area) was below 2%, and the inter-day precision was below 3%.

#### 4.2.4. Measurement of the Melatonin-Synthesis Pathway Enzymes Activities

##### Tryptophan Hydroxylase Activity

The activity of TPH was determined by measuring the accumulation of 5-HTP during incubation of the pineal homogenate with appropriate substrates and the AADC inhibitor [53]. Briefly, 20 μL of the homogenate was added to an Eppendorf tube containing 10 μL of 8 mM tryptophan, 10 μL of 3.2 mM tetrahydrobiopterin, 10 μL of 8 mM dithiothreitol, 10 μL of 8 mM ammonium iron (II) sulfate hexahydrate, 2.5 μL of catalase from bovine liver, 10 μL of 8 mM NSD 1015, and 7.5 μL of 1M TRIS acetate buffer. The tubes were incubated in a water bath at 38 °C for 30 min. The enzymatic reaction was stopped by adding 20 μL of 1 M perchloric acid. The tubes were kept in an ice-bath for 15 min and then centrifuged at 30,000× *g* (4 °C) for 15 min. To determine the blank values, the homogenates were added to the tubes containing 20 μL of 1 M perchloric acid and the above mentioned reagents. The tubes were incubated in an ice-bath for 30 min and centrifuged. Supernatants were carefully transferred into HPLC autosampler vials. The chromatographic analysis was performed as described for indoles, except that the detector sensitivity was set to level 2. The injection volume was 10 μL. The intra-day precision (RSD of peak area) and the inter-day precision were below 2%.

##### Aromatic L-Amino Acid Decarboxylase Activity

The activity of AADC was determined by measuring decarboxylation of 5-HTP into 5-HT in the presence of monoamine oxidase inhibitor [54]. The pineal homogenate (20 μL) was added to an Eppendorf tube containing 10 μL of 5 mM 5-HTP, 10 μL of 0.05 mM pyridoxal 5’-phosphate in phosphate buffer (0.2 M, pH 7.8), and 10 μL of 5 mM pargyline in the same buffer. After incubation at 38 °C for 30 min., the reaction was stopped by adding 10 μL of 1 M perchloric acid, the tubes were incubated in an ice-bath for 15 min, centrifuged, and the supernatant was transferred into an HPLC autosampler vial.

The chromatographic analysis was performed using the system, column, and mobile phase described in Section 4.2.3. The flow rate of the mobile phase was 1 mL/min. The methanol concentration was increased from 5% (*v/v*) to 10% (*v/v*) during the first 7 min of separation, kept at a constant level for 2 min, and then increased to 30% (*v/v*) during 11 min. After 10 min, the methanol level was stepwise decreased to the initial value. The injection volume was 2.5 μL. The detection parameters were as follows: an excitation wavelength 280 nm, an emission wavelength 345 nm, and temperature 38 °C. The detection was turned on at the 6th min of separation, and the detector sensitivity was set to level 1. The retention time of 5-HT was 6.4–6.5 min. The intra-day precision and the inter-day precision were below 2%.

##### Arylalkylamine N-Acetyltransferase Activity

The AA-NAT activity was determined by measuring the accumulation of N-acetyltryptamine [55]. The pineal homogenate (20 μL) was added to an Eppendorf tube containing 10 μL of 5 mM 5-tryptamine, 10 μL of 2.55 mM acetylo-CoA, and 10 μL of 0.2 mM phosphate buffer pH 6.8. The incubation and termination of the enzymatic reaction were performed as described above.

The chromatographic analysis was performed as described for AADC activity, except that the fluorescence detection was turned on at the 18th min of separation, and the detector sensitivity was set to level 4. The injection volume was 5 μL. The retention time of N-acetyl tryptamine was 25.9–26.0 min. The intra-day precision and inter-day precision were below 2%.

##### N-Acetylserotonin O-Methyltransferase Activity

The ASMT activity was determined by measuring the accumulation of MLT [56]. The pineal homogenate (20 μL) was added to an Eppendorf tube containing 10 μL of 2 mM N-acetylserotonin in phosphate buffer (0.2 M, pH 7.8) and 10 μL of 0.4 mM S-adenozynomethionine in phosphate buffer (0.2 M, pH 7.8). The incubation and termination of the enzymatic reaction were performed as described above.

The chromatographic analysis was performed as described for AA-NAT activity. The injection volume was 5 μL. The retention time of N-acetyl tryptamine was 25.0–25.1 min. The intra-day precision and inter-day precision were below 2%.

### 4.3. Statistical Analysis

The data were analyzed by two-way analysis of variance (ANOVA) with the light conditions and the sampling time as factors. Duncan’s test was used as a post hoc procedure. A *p*-value < 0.05 was considered as significant. The analyses were performed using Dell Statistica 13 (Version 13.1 PL, Dell Inc., Tulsa, OK, USA).

## 5. Conclusions

The profile of pineal indole metabolism in 4-week-old turkeys is characterized by (i) high-amplitude rhythms in AA-NAT activity and the contents of NAS and MLT, (ii) equal relative amounts of 5-HT and 5-HIAA, and (iii) higher content of MLT than NAS. This profile differs markedly from that in the chicken by the intensity of 5-HT oxidative deamination and the mechanisms responsible for the generation of 5-MIAA and 5-MTOL rhythms, and from those in the goose and duck pineal organs by the amplitudes of NAS and MLT rhythms and the proportion of NAS to MLT. These data strongly support the idea that metabolism of pineal indoles shows important species-specific features.

The prominent daily rhythmicity is maintained in the pineal organs of turkeys kept during the photophase under red, blue, and green light, even at the intensity of 16 lx. However, the monochromatic light significantly modifies the pineal indole metabolism and its effects are dependent on the color and intensity of light. The pronounced changes occur during the course of daily rhythm of MLT synthesis. At the photophase onset, blue light has stronger inhibitor effects on MLT synthesis than green and red light, and the inhibitory effect of monochromatic light increases with its intensity, independent of wavelength. Exposition to blue light during the photophase results in slower increase in AA-NAT activity at night compared to red and green lights with the same intensity. The increase in MLT synthesis after the onset of darkness is also dependent on the intensity of monochromatic light during the photophase. The color and intensity of light affect the time of nocturnal peak occurrence and the amplitude of daily changes in the AA-NAT activity and contents of NAS and MLT. Moreover, red light significantly increases the synthesis of 5-HT, which results in higher levels of 5-HIAA, 5-HTOL, 5-MIAA, and 5-MTOL. Further studies are needed to explain the mechanisms of spectral-dependent action of light on the pineal indole metabolism.

## Figures and Tables

**Table 1:** Light conditions during the last 7 days of the experiment.

Part of the Experiment	Animal Room	Light Source	Light Intensity
Red color	RL	GOQ 3 LED Red Dominant wavelength: 620–626 nm Luminous flux of module 24 lm Luminous intensity of LED: 1600–2200 mcd Half angle ± 60°	16 lx
	RM		32 lx
	RH		64 lx
	W	Tungsten lamps, 3200 K	16 lx
Blue color	BL	GOQ 3 LED Blue Dominant wavelength: 450–460 nm Luminous flux of module 21 lm Luminous intensity of LED: 600–1200 mcd Half angle ± 60°	16 lx
	BM		32 lx
	BH		64 lx
	W	Tungsten lamps, 3200 K	16 lx
Green color	GL	GOQ 3 LED Green Dominant wavelength: 520–530 nm Luminous flux of module 48 lm Luminous intensity of LED: 3000–4000 mcd Half angle ± 60°	16 lx
	GM		32 lx
	GH		64 lx
	W	Tungsten lamps, 3200 K	16 lx

## Abbreviations

5-HIAA	5-hydroxyindoleacetic acid
5-HT	serotonin
5-HTOL	5-hydroxytryptophol
5-HTRP	5-hydroxytryptophan
5-MIAA	5-methoxyindoleacetic acid
5-MTAM	5-methoxytryptamine
5-MTOL	5-methoxytryptophol
5-MTRP	5-methoxytryptophan
AADC	aromatic L-amino acid decarboxylase
AA-NAT	arylalkylamine N-acetyltransferase
ASMT	N-acetylserotonin O-methyltransferase
HPLC	high pressure liquid chromatography
MLT	melatonin
NAS	N-acetylserotonin
TPH	tryptophan hydroxylase
TRP	tryptophan
BL	blue light 16 lx
BM	blue light 32 lx
BH	blue light 64 lx
GL	green light 16 lx
GM	green light 32 lx
GH	green light 64 lx
RL	red light 16 lx
RM	red light 32 lx
RH	red light 64 lx
W	white light 16 lx

## Appendix A

Indole metabolism (Figure A1) in the pineal organ begins with the uptake of tryptophan (TRP) into pinealocytes. TRP is hydroxylated by tryptophan hydroxylase to 5-hydroxytryptophan (5-HTRP), which is decarboxylated by aromatic amino acid decarboxylase to serotonin (5-HT). This indoleamine is a starting point for three metabolic pathways. The first one is the transformation of 5-HT into melatonin (MLT). This process includes two steps: acetylation by arylalkylamine N-acetyltransferase (AA-NAT) to N-acetylserotonin (NAS), and methylation by N-acetylserotonin O-methyltransferase (ASMT) to MLT. The second one is oxidative deamination of 5-HT, which results in the formation of 5-hydroxyindoleacetic acid (5-HIAA) and 5-hydroxytryptophol (5-HTOL). These indoles are methylated by ASMT to 5-methoxyindoleacetic acid (5-MIAA) and 5-methoxytryptophol (5-MTOL). Moreover, 5-HT can be methylated by ASMT to 5-methoxytryptamine (5-MTAM). The putative pathway is the methylation of 5-HTRP into 5-methoxytyptophan (5-MTRP)
